# 5,8-Dimethyl-9*H*-carbazole Derivatives Blocking hTopo I Activity and Actin Dynamics

**DOI:** 10.3390/ph16030353

**Published:** 2023-02-25

**Authors:** Jessica Ceramella, Domenico Iacopetta, Anna Caruso, Annaluisa Mariconda, Anthi Petrou, Athina Geronikaki, Camillo Rosano, Carmela Saturnino, Alessia Catalano, Pasquale Longo, Maria Stefania Sinicropi

**Affiliations:** 1 Department of Pharmacy, Health and Nutritional Sciences, University of Calabria, 87036 Arcavacata di Rende, Italy; 2 Department of Science, University of Basilicata, 85100 Potenza, Italy; 3 Department of Pharmacy, School of Health, Aristotle University of Thessaloniki, 54124 Thessaloniki, Greece; 4 U.O. Proteomica e Spettrometria di Massa, IRCCS Ospedale Policlinico San Martino, Largo R. Benzi 10, 1632 Genova, Italy; 5 Department of Pharmacy-Drug Sciences, University of Bari “Aldo Moro”, 70126 Bari, Italy; 6 Department of Chemistry and Biology, University of Salerno, Via Giovanni Paolo II, 132, 84084 Fisciano, Italy

**Keywords:** human topoisomerases I/II, anticancer, docking simulation, actin dynamics

## Abstract

Over the years, carbazoles have been largely studied for their numerous biological properties, including antibacterial, antimalarial, antioxidant, antidiabetic, neuroprotective, anticancer, and many more. Some of them have gained great interest for their anticancer activity in breast cancer due to their capability in inhibiting essential DNA-dependent enzymes, namely topoisomerases I and II. With this in mind, we studied the anticancer activity of a series of carbazole derivatives against two breast cancer cell lines, namely the triple negative MDA-MB-231 and MCF-7 cells. Compounds **3** and **4** were found to be the most active towards the MDA-MB-231 cell line without interfering with the normal counterpart. Using docking simulations, we assessed the ability of these carbazole derivatives to bind human topoisomerases I and II and actin. *In vitro* specific assays confirmed that the lead compounds selectively inhibited the human topoisomerase I and interfered with the normal organization of the actin system, triggering apoptosis as a final effect. Thus, compounds **3** and **4** are strong candidates for further drug development in multi-targeted therapy for the treatment of triple negative breast cancer, for which safe therapeutic regimens are not yet available.

## 1. Introduction

**Figure 1:**
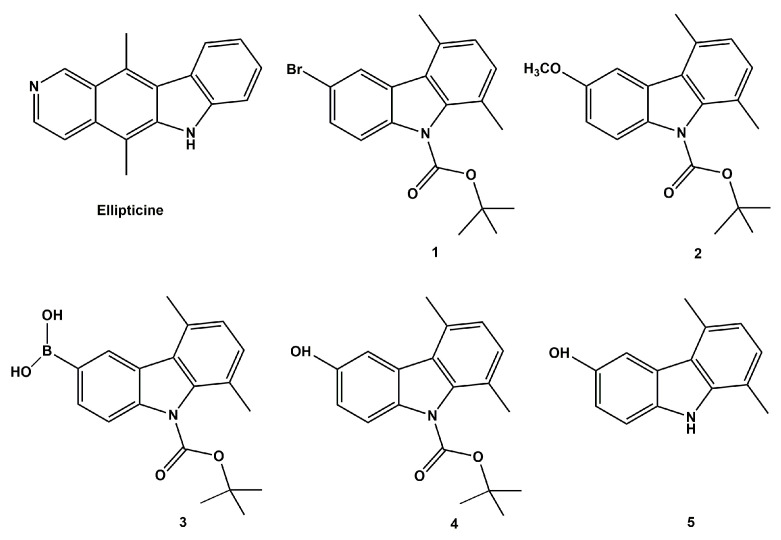
Structures of Ellipticine and the studied carbazole derivatives (**1**–**5**) previously synthesized by us [25].

One of the principal targets of pharmaceutical research is represented by the design of new and valid anticancer drugs characterized by higher selectivity on neoplastic cells and lower toxicity on normal ones. Over the years, medical and pharmaceutical researchers’ attention has been focused on the carbazole scaffold, which is present in important classes of indole-containing heterocycles characterized by widespread biological activities, including anticancer, antibacterial, antiviral, antioxidant, antidiabetic, and neuroprotective ones [1]. These characteristics resulted in the extensive applications of carbazole derivatives in the field of medicinal chemistry [2,3]. The carbazole skeleton is the key structural motif of many synthetic and natural biologically effective molecules acting as DNA intercalating agents and able to interfere with the activity of crucial enzymes involved in cancer progression, such as the topoisomerases (Topos) [4]. The latter are ubiquitous enzymes, vital for gene expression, chromosome segregation, as well as DNA replication and recombination, due to their capability to solve DNA supercoiling by cutting one or both strands of the DNA duplex [5]. Based on their mechanism of action, two classes of DNA topoisomerases have been identified from eukaryotes: the DNA topoisomerase I (Topo I) which acts by making a temporary break in one strand of DNA, while the DNA topoisomerase II (Topo II) mediates the ATP-dependent DNA double-strand breaks [6]. Anticancer agents targeting both Topo I and II have been proven to be highly effective in cancer treatment [7]. Because of these interesting properties, extensive research concerning the chemistry and biological activities of carbazoles targeting topoisomerases has been reported since the characterization of the 9*H*-carbazole in 1872 by Graebe and Glaser [1,8,9,10]. Ellipticine (Figure 1) is one of the first studied natural occurring alkaloids with a carbazole nucleus and several studies have evidenced its wide biological effectiveness, highlighting its capability to act as DNA intercalating and Topos inhibitor [11]. However, Ellipticine is also characterized by a high toxicity. Thus, to obtain more active and safer derivatives, great efforts in the design and synthesis of carbazole analogues have been made over the last years. For instance, new pyrrolo[2,3-α]carbazole derivatives were found to significantly reduce the Topo I activity in a concentration dependent manner [12], while some 11*H*-pyrido[*a*]carbazole ones resulted good DNA intercalating and Topo II inhibitors [13]. Moreover, a new series of racemic and chiral carbazole aminoalcohols was proved to possess a potent Topo I inhibitory activity [14]. Two Ellipticine analogues acted as good inhibitors of human topoisomerase II (hTopo II) and resulted more potent than the reference molecule. In our previous works, more than one series of carbazole compounds have been designed and prepared. In particular, the new synthesized *N*-thioalkylcarbazole, *N*,*N*,*N*-trimethylethanammonium iodide alkylcarbazole, and 1,4-dimethylcarbazole derivatives exhibited exciting cytotoxic profiles on different breast cancer cell lines and inhibited the hTopo II decatenation activity [15,16,17]. Moreover, we demonstrated that some benzothienoquinazolinones carbazole bioisosters [18] exerted good anticancer activity on breast cancer cells due to their capability to inhibit the hTopo I supercoil relaxing activity. Beyond that, the benzothienoquinazolinones carbazole bioisoster also targeted the microtubule network involved in vital cellular functions, such as mitosis, cell migration, and cell signaling. These findings are seminal in the fight against breast cancer, which still represents the main cancer-related cause of disease for women, and its incidence and mortality have risen worldwide in recent years [19]. Over the years, numerous carbazole derivatives were designed, synthesized, and examined for their anti-breast cancer activity [20,21,22]. Recently, Vlaar et al. studied new analogues of EHop-016, a carbazole compound acting as a Rac1 inhibitor, implicated in the intracellular actin polymerization. Some of them exerted good antiproliferative activity on different breast cancer cells lines and inhibited the migration process in the metastatic MDA-MB-231 cells. In addition, the lead compound improved by approximately four-fold *in vitro* efficacy in inhibiting the activity of the Rho GTPase Rac1 in both MDA-MB-231 and MDA-MB-435 cell lines if compared with EHop-016 [23]. Moreover, Butler-Fernández et al. published a new series of *N*-alkyl-3,6-dibromocarbazole and *N*-alkyl-5-bromoindole derivatives, whose anticancer and anti-migratory effects in MCF-7 and MDA- MB-231 breast cancer cell lines are connected to the interference with the intracellular actin dynamics [24].

Building upon these findings, the goal of this work was to evaluate the anticancer activity of a series of five carbazole derivatives (**1**–**5**) (Figure 1), previously synthesized by us [25], against two human breast cancer cell lines, namely MCF-7 and MDA-MB-231. Docking studies and *in vitro* assays were performed in order to elucidate the mechanism of their action, revealing that the two individuated leads target hTopo I and actin. Finally, they can be considered promising candidates for the development of new multi-target agents in the treatment of triple negative breast cancer.

## 2. Results

### 2.1. Chemistry

Carbazole derivatives (**1**–**4**) were synthesized as reported in the literature [25]. The synthesized molecules (**1**–**4**) differ by the substituent in position 6, while compound **5** is analogous to compound **4,** but does not bear the Boc group. The substituents selected for the design of the molecules were: -Br, -OCH_3_, -B(OH)_2_ e -OH, whose presence allowed to obtain valuable information about the correlation between the architecture of the molecules and the biological activity.

### 2.2. Effects on Breast Cancer and Normal Cells Viability

The inhibition of cancer cell growth exerted by the five considered compounds (**1**–**5**) has been evaluated *in vitro* against two models of breast cancer, namely the estrogen receptor positive (ER+) MCF-7 and the triple negative MDA-MB-231 human breast cancer cells (lacking ER, PR, and HER-2/Neu amplification), together with the normal counterpart, namely the non-malignant breast epithelial cells MCF-10A. The IC_50_ values were determined at 72 h after treatment by means of the MTT assay and are reported in Table 1. As reference molecule, we adopted Ellipticine, the parent molecule with a carbazole scaffold from which they have been derived. Our results indicated that, among the carbazole derivatives, compound **4** resulted the most active, since it drastically reduced the growth of the MDA-MB-231 cells with an IC_50_ value of 0.73 ± 0.74 μM. Then, compound **3** also exhibited good anticancer activity against the triple negative cancer cells, with an IC_50_ value of 1.44 ± 0.97 μM. A moderate activity was noticed against the MCF-7 cells, with compound **4** being most active. More interestingly, compound **4** did not affect the MCF-10A cells viability, until the concentration of 100 μM, whereas compound **3** induced the death of the half of cells at a concentration of approximately 52 μM. Compounds **5**, **2,** and **1** exhibited decreasing anticancer activity against the MDA-MB- 231 cells, with IC_50_ values equal to 6.59 ± 0.68, 8.19 ± 0.26, and 43.45 ± 1.21 μM, respectively. Moreover, no effects were recorded against the MCF-7 cells, at least until the concentration of 100 μM. These outcomes suggest that the presence of the bromine substituent on the carbazole nucleus is responsible of the net decrease of the activity, whereas the hydroxy and boron substituents seem to be determinant for the observed higher activity. It is important to highlight that compounds **3** and **4** possess a higher anticancer activity against the MDA-MB-231 cells and a better cytotoxic profile than the Ellipticine, which is not even selective amongst breast cancer and normal cells. Thus, the two lead compounds were chosen for subsequent studies aiming at individuating the molecular mechanisms underlying the observed anticancer activity.

### 2.3. Docking Studies

**Figure 2:**
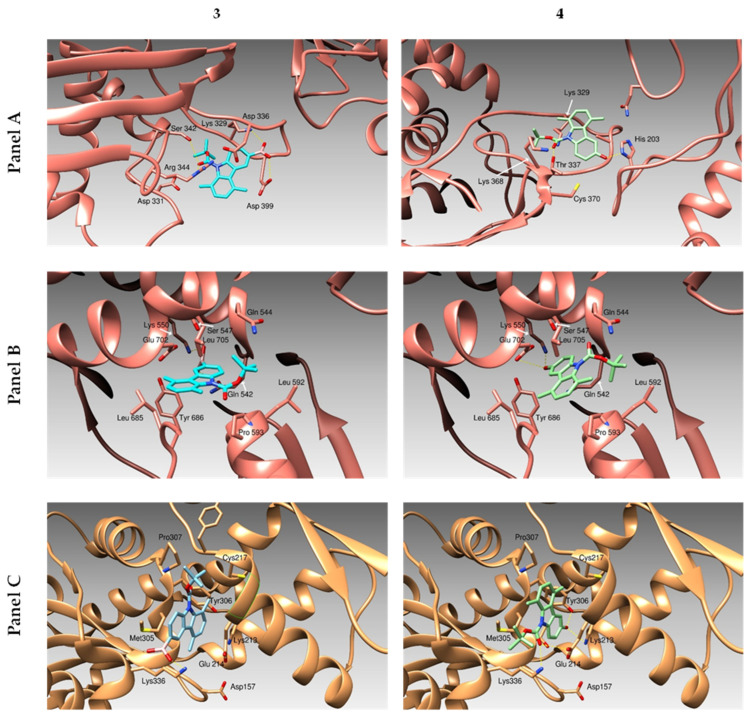
The three-dimensional structure of the human proteins Topoisomerase I (Panel A), Topoisomerase II (Panel B) and Actin (Panel C) bound to compounds **3** and **4** are drawn. Proteins are schematically reported as ribbons. Ligands binding poses are described as colored sticks.

Molecular docking simulations were performed in order to understand the possible binding modes of the compounds described above (Figure 1) and the target proteins, namely the actin and the human Topoisomerases I and II. We considered a “blind-docking approach” for our simulations: no “a priori” information about the binding site was provided to the system. This kind of procedure has been successfully used by our research group in several other studies (some examples in [26,27,28,29]). Using this kind of approach, we aimed both to identify the most promising candidate among our compounds and to further improve the atomic structure of our molecules in order to design and synthesize better lead compounds. Our study was based on the compound binding affinity to the two human Topoisomerases and actin (Table 2), as calculated by the program Autodock (this program calculates a binding affinity constant Ki based on the binding Energy, according to the expression Ki = exp (deltaG/(R * T)). To discriminate the successful candidates, we took into consideration the clusterization of the results from the simulations, as discussed in previous work [30]. Eventually, the obtained binding mode was examined to evaluate the quality of the protein–ligand interactions. Docking simulations (Figure 2 and Figure S1, panels C) suggested our compounds were able to dock actin and the hTopo I and II, forming several hydrogen and hydrophobic interactions. Particularly, our molecules dock the actin in a protein cleft occupied by the Latrunculin B in the crystal structure described by Wang et al. [31]. The actin residues Asp 157, Lys 213. Glu 214, Thr 303, and Tyr 306 are involved in the hydrogen bonding to our compounds. The protein cleft is completed by the hydrophobic residues Ala 181, Leu 185, Leu 216, Cys 217, Pro 307, and Ile 309 that contribute to stabilize the compounds binding. Simulations carried over the hTopo I, as the protein target, identified three distinct possible binding sites for our compounds. Compound **5** (Figure S1, panel A) binds a first site forming hydrogen bonds with Asp 331 and hydrophobic interactions with the backbone of the loop Asn 327- Gly 339 and the side chains of residues Ala 334 and Val 338. In the same pose, compound **3** (Figure 2, panel A) forms additional hydrogen bonds with residues Lys 329, ser 342 Arg 344, and Asp 399. A second binding lays on the opposite site of the loop Asn 327- Gly 339 and hosts compounds **4** and **1** (Figure 2 and Figure S1, panels A, respectively). These compounds are both stabilized by hydrophobic interactions with Ala 334 and Thr 337. Compound **4** forms hydrogen bonds with His 203, Lys 329, Thr 337, and Lys 368, while compound **1** loses the interaction with His 203 but adds a halogen bond between its bromide moiety and Ser 342. On the other hand, compound **2** (Figure S1, panel A) docks to a protein area that is far from the previously described sites. In this case, compound **2** is surrounded by hydrophobic residues Leu 321, the aromatic ring of Tyr 373, Val 377, Ile 464, and Val 550, forming hydrogen bonds with Asn 467 and Gln 469. The same molecules described above bind the hTopo II close to the DNA-gate (Figure 2 and Figure S1, panels B). The residues Gln 726 and Asn 851 are hydrogen-bonded to compound **5** (Figure S1, panel A), Gln 544 and Lys 550 to compound **3** (Figure 2, panel A), and Gln 544, Lys 550, and Gln 542 to compound **4** (Figure S1, panel A). Due to the presence of a methyl group, compound **2** (Figure S1, panel A) loses the ability to form hydrogen bonds with Gln 544, Lys 550, and Gln 542 (those three molecules are almost superposed), whilst compound **1** (Figure S1, panel A) makes a halogen bond with Arg 672. The pocket is contoured by hydrophobic residues Leu 592, Pro 593 Leu 705, Leu 685, and Tyr 686.

### 2.4. Inhibition Assays on Human Topoisomerase I and II

**Figure 3:**
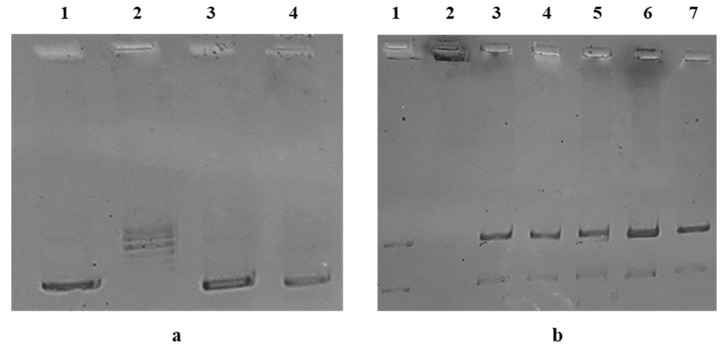
(**a**) hTopo I supercoiled relaxing activity. hTopo I was exposed to the vehicle alone (DMSO, lane 2) or compounds **3** and **4** at the concentration of 1 μM (lanes 3 and 4). Then, the hTopo I reaction products were visualized on agarose gel. Supercoiled DNA (plasmid pHOT1) was used as marker (lane 1). (**b**) hTopo II decatenation assay. hTopo II was exposed to the vehicle alone (DMSO, lane 3) or compounds **3** and **4** at the concentrations of 1 μM (lanes 4 and 5) and 10 μM (lanes 6 and 7). Then, the hTopo II reaction products were visualized on agarose gel. Decatenated DNA and kinetoplast DNA (kDNA) were used as markers (lanes 1 and 2).

The carbazole derivatives **1**–**5** were tested for their ability to inhibit the human Topoisomerase I (hTopo I) and II (hTopo II), using specific enzymatic assays, as reported in [17,18], and the obtained results are shown in Figure 3.

First, only **3** and **4** totally inhibited the hTopo I supercoiling relaxing activity at the concentration of 1 μM. Indeed, as shown in Figure 3a (lanes 3 and 4), a clear band of the uncut plasmid DNA at the bottom of the gel is present. The uncut plasmid pHOT1, used as marker, is present in Figure 3a, lane 1. On the contrary, in the control reaction (only vehicle, Figure 3a, lane 2), it is possible to observe the presence of multiple bands corresponding to the relaxed plasmid DNA, used as substrate.

Next, we screened all the compounds for their activity against the hTopo II but, in this case, none of the tested compounds have been able to block the decatenation activity at the concentration of 1 µM (Figure 3b, lanes 4 and 5). Moreover, since **3** and **4** totally blocked the hTopo I, in order to exclude a dose-dependent activity, the concentration was increased to 10 μM (see Figure 3b, lanes 6 and 7). Again, no inhibitory activity was recorded. Indeed, two bands related to the DNA decatenation products are present at the bottom of the agarose gel, which denote the enzyme full activity, both in the control (only vehicle, Figure 3b, lane 3) and the tested compound reactions (Figure 3b, lanes 4–7). Thus, we can conclude that the lead compounds **3** and **4** are selective inhibitors of the hTopo I, being inactive against the hTopo II.

### 2.5. Influence of Compounds ***3*** and ***4*** on Actin Dynamics

**Figure 4:**
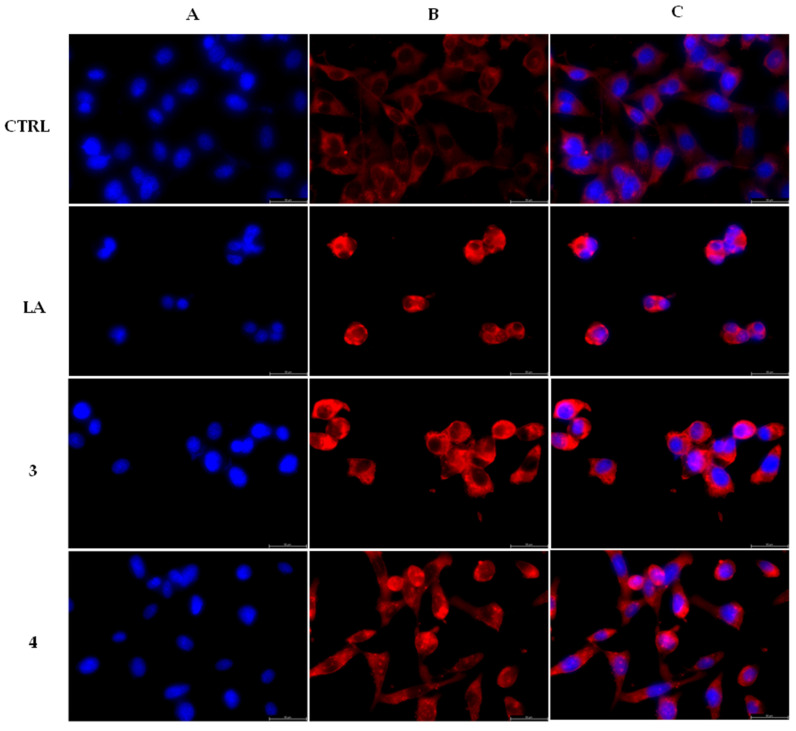
Actin immunofluorescence studies. MDA-MB-231 cells were exposed for 24 h to the vehicle alone (CTRL), 0.1 μM LA or carbazole derivatives **3** and **4** (used at their IC_50_ values). Then the cells were further processed, as indicated in the experimental section. The inverted fluorescence microscope was adopted to observe and image all the immunofluorescence figures (40× magnification). Panels A: nuclear stain with DAPI (λ_ex/λem_ = 350/460 nm); Panels B: β-actin (Alexa Fluor^®^ 568; λ_ex/λem_ = 644/665 nm); Panels C show a merge. Representative fields are reported.

In order to determine whether the lead compounds **3** and **4** may effectively regulate the actin system, we employed both the immunofluorescence and *in vitro* direct enzymatic assays. Thus, MDA-MB-231 were treated for 24 h with compounds **3** and **4** at a concentration equal to their IC_50_ values, respectively. As negative and positive controls, we used the only vehicle (DMSO) and latrunculin A (LA) at a concentration of 0.1 µM, respectively. After processing, the cells were observed under a fluorescent microscope, (see experimental section for the details). Our outcomes showed that the actin filaments are regularly organized in the cell cytoplasm in the DMSO treated cells (Figure 4, CTRL, panel B), whereas under the LA exposure (Figure 4, LA, panel B), the cells underwent an important shape change, appearing circular, because of the interference with the actin system. Moreover, the latter looked brighter and stocked in the cytoplasm in dot-like structures or thicker fibers. Again, the MDA-MB-231 cells, under exposure to compounds **3** and **4**, lost their shape (Figure 4, compounds **3** and **4**, panels B), as we observed under LA treatment, and with a very similar arrangement of the actin network. These outcomes suggest that compounds **3** and **4** interfere with the normal actin organization in MDA-MB-231 cells, with a similar behavior of LA.

**Figure 5:**
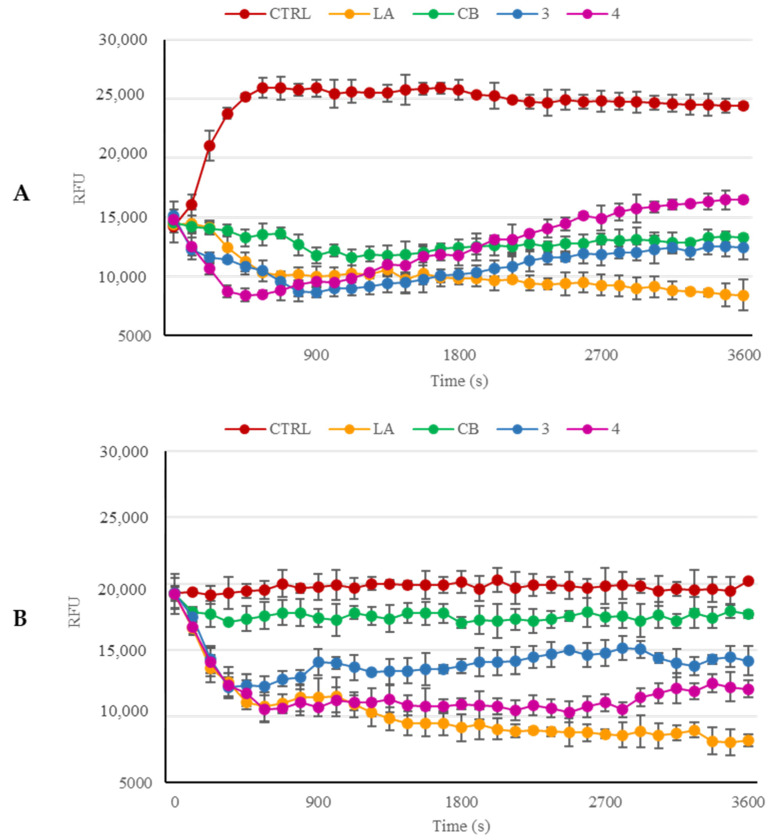
(**A**) *In vitro* actin polymerization assay. Compounds **3** and **4** (used at the concentration of 5 µM) were incubated with the labeled rabbit muscle actin in order to verify their ability to inhibit the protein polymerization. (**B**) *In vitro* actin depolymerization assay. After actin polymerization, compounds **3** and **4** (5 µM) were added to the reaction mixture, in order to determine their ability to act as depolymerizing agents. For both the assays, the vehicle DMSO was used as a negative control. Actin-targeting agents, LA and CB, both at the concentration of 5 μM, were used as positive controls. The assemblage of the actin filaments was established by monitoring the fluorescence (λ_Ex/Em_= 365/410 nm) in kinetic mode for 1 h at room temperature by using a microplate reader. The graphics are representative of three separate experiments and error bars represent the standard deviations.

With the aim to confirm the immunofluorescence results and substantiate whether compounds **3** and **4** could inhibit the actin polymerization mechanism and/or boost the F-actin depolymerization, we employed a fluorescent-labeled purified rabbit actin. LA and cytochalasin B (CB) were used as positive controls for both the polymerization inhibition and F-actin subunits dissociation, or only the polymerization inhibition, respectively. In the negative control reaction (only vehicle), actin monomers undergo a normal polymerization, as visible in Figure 5, panel A. The reaction curve rises in approximately 5 min to a value of about 25,000 RFU (plateau) which remains almost unvaried until the end of the experiment. Contrariwise, the two reference molecules, i.e., LA and CB, used at the concentration of 5 µM, braked the actin polymerization, mostly LA rather than CB, under the adopted experimental conditions, and the LA curve dropped until a value of about 8000 RFU at the reaction end. The CB curve, similar to that of LA, showed a decrease and ended at approximately 13,000 RFU.

Lastly, compounds **3** and **4**, used at the concentration of 5 µM, both exhibited an inhibitory effect against actin polymerization, but with a lesser efficacy than LA. However, compound **3** curve was lower than that of CB and reached a final value of about 13,000 RFU, which is pretty similar to that of CB, whereas the final value of compound **4** was around 16,000 RFU. Thus, compound **3** has an inhibitory effect slightly superior to that of CB throughout the whole reaction, whereas compound **4** seemed to inhibit better the actin polymerization than CB only in the first 40 min.

Moreover, we performed the F-actin depolymerization assay to verify whether compounds **3** and **4** could induce the actin depolymerization as well as the LA does (Figure 5, panel B). For this purpose, we started the actin polymerization for one hour under the same experimental conditions used previously. Then, we added the compounds or the reference molecules at the concentration of 5 µM and monitored the reactions for an additional hour. Our results indicated that the LA induced an important fall of the curve until a final value of about 8000 RFU, indicating the F-actin depolymerization. On the contrary, the control polymerization reaction (only vehicle) exhibited an almost constant value (20,000 RFU) until the reaction end. The addition of CB was not able to produce the same effect of LA. Instead, its behavior follows that of the vehicle, suggesting that CB does not induce the F-actin depolymerization. Contrarily, both the compounds were able to induce an important depolymerizing effect on the F-actin, most evident in the first 7 min after the exposure. Particularly, compounds **3** and **4** produced final values of approximately 14,000 and 12,000 RFU, respectively. Altogether, our outcomes suggest that compounds **3** and **4** produced a similar effect to that of LA on actin, even if to a lesser extent, inducing the inhibition of the polymerization reaction and, at the same time, accelerating the dissociation of F-actin. This combined effect leads to a disorganization of the intracellular actin network.

### 2.6. Compounds ***3*** and ***4*** Trigger Apoptosis in MDA-MB-231 Cells

**Figure 6:**
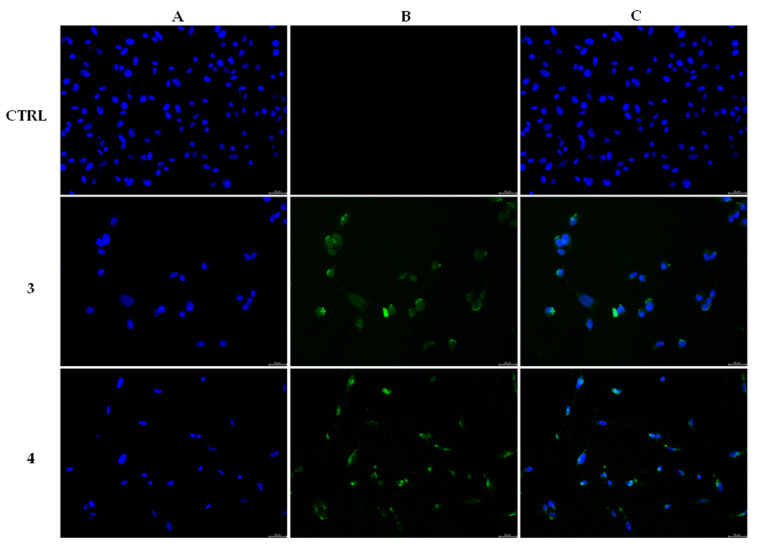
Tunel Assay. MDA-MB-231 breast cancer cells were treated with compounds **3** and **4** at the concentration equal to their IC_50_ values or with vehicle (CTRL) for 24 h. Then they were exposed to the TdT enzyme, further processed (see experimental section for more details) and visualized under a fluorescence microscope (20× magnification). Panels A: DAPI, λ_ex/em_= 350 nm/460 nm. Panels B: CF^TM^488 A, λ_ex/em_ = 490 nm/515 nm. Panels C show the overlay channels.

Having individuated two intracellular targets, we wondered whether the lead compounds could induce apoptosis in MDA-MB-231 cells. Thus, we performed a TUNEL assay. The cells were treated and processed as described in the experimental section (Section 4.2.6) and the obtained outcomes, shown in Figure 6, suggested that both the compounds are able to trigger apoptosis in MDA-MB- 231 cells. Indeed, the exposure to both compounds produced a green nuclear fluorescence, already at 24 h, in MDA-MB-231 cells (Figure 6, **3** and **4**, panels B, CF^TM^488A) as a consequence of the damaged DNA. This event did not happen in the DMSO-treated cells (Figure 6, CTRL, panel B, CF^TM^488A), indicating the lack of a massive DNA break. The overlay channel (Figure 6, panels C) is also shown.

### 2.7. Druglike Properties, Toxicity and Drug-Likeness

‘Drug-like’ molecules were evaluated *in silico* for their ADMET profile in order to rapidly screen multiple properties [32]. Compounds that have been predicted to exhibit toxicity, high blood–brain barrier permeability, low water solubility, and poor Caco2-permeability were excluded from potential hits. The server pkCSM [33] was used for this purpose. pkCSM relies on graph-based signatures. These encode distance patterns between atoms in order to represent the small molecule and to train predictive models (Table S1).

Computational studies are considered a viable approach to drug discovery, and they have several advantages over in vivo studies, especially in reducing cost, time, and animals sacrifice. These approaches, nowadays, are broadly used in studies of the physicochemical and pharmacokinetic properties of compounds in medicinal chemistry. Lipinski’s rule of five (Ro5) is considered a standard for drug development [34], and violations of Ro5 are MW > 500, lipophilicity (LogPo/w) > 5, hydrogen bond determined for binding donors (HBD) < 5. Violations of these rules lead to reduced intestinal absorption, penetration, or solubility [35]. The next extension of the Lipinski Ro5 includes polar surface area (PSA < 140 Å^2^), which is an important predictor of drug oral bioavailability. 

**Figure 7:**
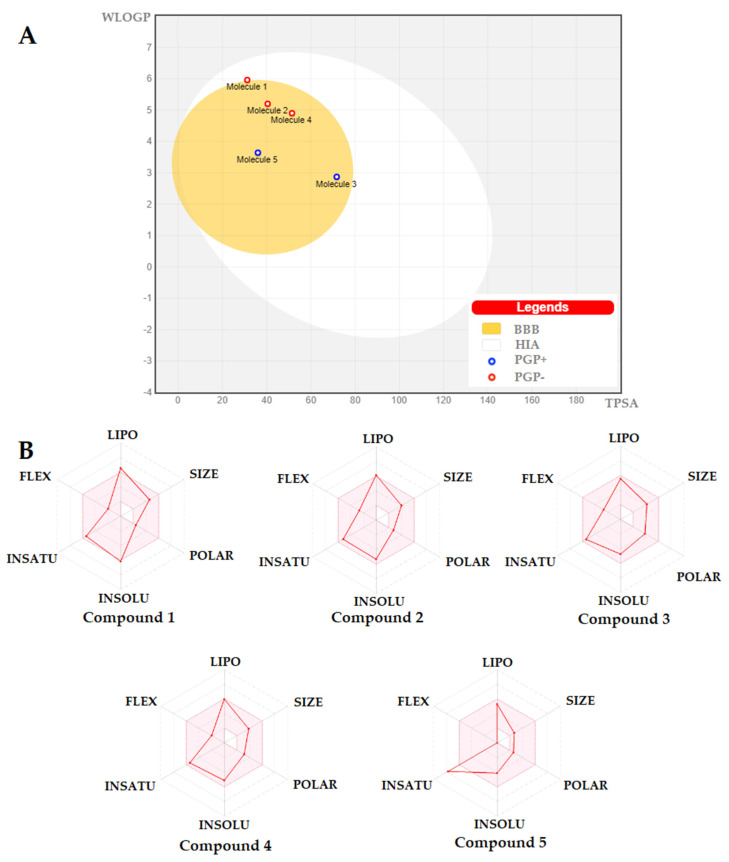
(**A**) BOILED-Egg diagram for all the designed compounds. (**B**) Bioavailability radar chart of all compounds. The pink area represents the optimal range for each property for oral bioavailability, Lipophilicity (LIPO): XLOGP3 between −0.7 and +5.0, Molecular weight (SIZE): MW between 150 and 500 g/mol, Polarity (POLAR) TPSA between 20 and 130 Å^2^, Solubility (INSOLU): log S not higher than 6, Saturation (INSATU): fraction of carbons in the sp3 hybridization not less than 0.25, and Flexibility (FLEX): no more than 9 rotatable bonds.

The synthesized compounds have been studied *in silico* using the Swiss ADME software [36].The drug-likeness and bioavailability scores of all tested compounds are shown in Table 3. According to prediction results, the bioavailability score of all compounds was approximately 0.55. Furthermore, all compounds displayed moderate to good drug-likeness scores, ranging from −0.43 to 0.52. The best in the in-silico prediction result was achieved for the most active compounds, **3** and **4** with a drug-likeness score of 0.52 and 0.42 (Figure 7, Table 3). Moreover, these compounds showed no violation in all rules. 

The BOILED-Egg allows for the evaluation of passive gastrointestinal absorption (GIA), brain penetration (BBB), and P-glycoprotein (P-gp) activity in the presence of the molecule. The white region of the “BOILED-egg” represents the high probability of passive absorption by the gastrointestinal tract, and the yellow region (yolk) the high probability of brain penetration. Moreover, the points are colored in blue if predicted as active effluxes by P-gp (PGP+) and in red if predicted as non-substrate of P-gp.

## 3. Discussion

It is noteworthy that breast cancer is the most frequently diagnosed malignant tumor and the second most deadly cancer in women. Many important developments in cancer prevention, early diagnosis, and treatment have been achieved, but the complex etiopathogenesis and the development of chemoresistance make the fight against breast cancer difficult to win. Consequently, scientific research has pursued different approaches, such as multitarget therapies able to reduce the cancer cells growth and invasion and exert very low effects on normal cells [37]. Despite the use of successful targeted and tailored therapies, many types of cancer, amongst them the triple negative breast cancers (TNBC), are still difficult to treat, mostly because of their heterogeneity and resistance onset [38]. A successful strategy in breast cancer therapy is based on the apoptosis induction in tumor cells acting on different pathways that are potential targets, with a minimal or null effect on the growth of the normal cells [39,40]. With this in mind, we studied the anticancer properties of a series of carbazole derivatives, (**1**–**5**), since our experience and a lot of studies from literature indicated their important antitumor activities, together with antibacterial, anti-inflammatory, and many other properties [8,20,41,42]. One of the first studied compounds with a carbazole scaffold was Ellipticine, a planar natural alkaloid, with known antitumor activities due to its ability to intercalate DNA and regulating several cell pathways with a multimodal effect [43]. Thus, we adopted two human breast cancer cell lines, namely MCF-7 and the most aggressive and metastatic MDA-MB-231, together with the normal counterpart, MCF-10A cells. The obtained viability data, resumed in Table 1, demonstrated a low to high activity in MDA-MBA cells and a moderate to null activity in MCF-7 cells, under the experimental conditions used in these assays. Particularly, the most active compounds in MDA-MB-231 cells were **3** and **4**, which also exhibited moderate activity in MCF-7 cells and a lack of cytotoxicity in the MCF-10A cells. It is worthwhile to highlight that both the compounds possess a better cytotoxic profile and selectivity with respect to the reference molecule, Ellipticine. The latter and its several synthetic derivatives possess a wide range of intracellular targets, whose exact mechanisms of action are not yet totally clear. However, our previous studies, and others from the literature, suggested a major role in DNA intercalation and inhibition of DNA topoisomerases [20,44,45]. These enzymes are implicated in the correct DNA metabolism and are the main target of numerous chemotherapeutics that produce irreversible genomic damages and cancer cell death [46]. The molecular docking approach gave us a prediction of interaction between our molecules and target proteins [47], which suggested that all the compounds could dock to the hTopo I and II in different sites and with a different mode. Particularly interesting is the case of the hTopo I, where compounds **1** and **5** dock to a loop involved into the protein dimerization processes, therefore creating some local rigidity and impairing the functional oligomerization of the complex. On the other side, compounds **3** and **4** are positioned in an area that is normally occupied by DNA, while compound **2** is located in a site that does not seem functional. Only compound **3** was found to dock to a site usually occupied by the DNA in the case of hTopo II. Next, we adopted direct inhibition assays, which indicated that only the two most active compounds, **3** and **4**, were able to block totally the hTopo I activity at a concentration of 1 μM, whereas no inhibition was recorder for the other compounds. On the contrary, neither at the same concentration nor rising up to 10 μM were compounds **3** and **4** (Figure 3), and the other ones as well, able to inhibit the hTopo II, differently from the predictive docking simulations. These results are in agreement with literature studies [12,18,48] reporting that different carbazole derivatives may block both the hTopos or, selectively, only one and induce cancer cells death. Amongst the targets of carbazole derivatives, cytoskeletal proteins have attracted the attention of many researchers, and some of them were found to provoke a net disorganization of the tubulin filaments and their accumulation around cell nuclei [22,49,50]. However, the literature is still lacking studies reporting the effects on the actin metabolism, with the exception, e.g., of a study on cell motility exerted by the carbazole derivative wiskostatin. The latter is a cell-permeable N-alkylated carbazole derivative found to be a selective inhibitor of actin filaments assembly [51]. It is known that the cell cytoskeleton is implicated in the cancer cell metastasis formation process, a dramatic phenomenon that causes many deaths for cancer. Particularly, the actin and many regulatory proteins are modified to allow the abnormal growth of cancer cells and the development of migratory properties [52,53]. Again, *in silico* and *in vitro* approaches allowed us to prove the effects on actin dynamics, which evidenced, overall, a net regulation of the actin cytoskeleton. Indeed, MDA-MB-231 cells treated with **3** or **4** change their normal morphology, because of the evident interference with intracellular actin, whose network appeared disorganized and forms bundles unevenly distributed in the cell cytoplasm. Moreover, the polymerization/depolymerization assays indicated a behavior similar to that of LA, instead of CB, which only blocks actin polymerization but not the opposite reaction, confirming what has already been observed. Finally, as expected, MDA-MB-231 cells, under exposure to compounds **3** or **4**, underwent cell death by apoptosis, recorded by means of the TUNEL assay. This ultimate effect is due to the observed hTopo I and actin filament formation inhibition necessary to sustain the uncontrolled cancer cells growth and progression. Drug-likeness has greatly impacted the most recent medicinal chemistry, which considers different molecular properties, such as hydrophobicity, size, flexibility, presence of various pharmacophores features, bioavailability, transport properties, and so on. Regarding this, the obtained scores indicated that the most active derivatives **3** and **4** do not violate any rules and are predicted to be orally active, making them the most promising compounds to be further developed.

## 4. Materials and Methods

### 4.1. Docking Studies

We built the three dimensional models of hTopo I and II, as previously described [30], using as templates the crystal structures of the hTopo I in covalent and noncovalent complexes with DNA (PDB code 1A35) [54] and of Topo IIα in complex with a short DNA fragment and etoposide (PDB Code 5gwk) [31]. The crystal structure of the complex formed between the Beta/Gamma-Actin with Profilin and the acetyltransferase AnCoA-NAA80 [55] (PDB code 6nbw) was also used as a target for the docking simulations. The structures of the tested compounds have been built and energy minimized using the program MarvinSketch (ChemAxon ltd, Budapest, Hungary). Autodock v.4.2.2. program suite [56] was employed to evaluate the possible binding modes and the binding energies of our compounds to the above mentioned proteins. We chose to adopt a “blind docking” strategy for our simulations: the docking of the compounds to the different targets were done without any a priori knowledge of the binding site by the system. All the simulations were performed adopting the standard program default values. The protein and the ligands were prepared using the ADT graphical interface [57]. For each protein, polar hydrogens were added, Kollman charges assigned, and solvation parameters calculated. While the ligands were considered as fully flexible objects, each protein was considered as full rigid. To properly calculate affinity maps, a searching grid was extended all over the protein and the search was carried out using a Lamarckian genetic algorithm. Using this protocol, a population of 100 individuals with a mutation rate of 0.02 was evolved for 100 generations and the final evaluation of the results was conducted, listing the different poses of each molecule accordingly to its predicted binding energy. Further on, an analysis cluster based on root mean squares deviation (RMSD) values of each pose with respect to the starting geometry was performed. The lowest energetic conformation of the most populated cluster was considered as the best candidate. In case two or more clusters were almost equipopulated and their energy distribution was spread, the corresponding were considered as bad ligands [26]. The docking poses resulting from our simulations were ranked in order of their binding energy values and clustered on the basis of a RMSD cut-off value of 2.0 Å. From the structural analysis of the lowest energy solutions of each cluster, we could spot the protein binding site. Figures were drawn using the program Chimera [58].

### 4.2. Biology

#### 4.2.1. Cell Cultures

The used cell lines (MCF-7, MDA-MB-231 and MCF-10A) were obtained from American Type Culture Collection (ATCC, Manassas, VA, USA) and cultured as already indicated [59].

#### 4.2.2. MTT Assay

MTT assays (Sigma Aldrich (St.Louis, MO, USA)) were employed to evaluate the *in vitro* anticancer activities of all the studied compounds, as previously described [59]. The compounds were tested at different concentrations (0.1-1-10-25-50-100 μM) for 72 h. The IC_50_ values were calculated from the percent (%) of control using GraphPad Prism 9 (GraphPad Software, La Jolla, CA, USA).

#### 4.2.3. hTopo I Relaxation Assay and hTopo II Decatenation Assay

hTopo I relaxation assays were performed as indicated in the manufacturer’s protocol (TopoGEN, Port Orange, FL, USA) with some revisions [15]. hTopo I relaxation assays were performed in a final volume of 20 μL: 0.25 μg of supercoiled pHOT1 in TE buffer [TE: 10 mM Tris-HCl (pH 7.5), 1 mM EDTA] was added to a solution containing water, 1× assay buffer (10 mM Tris-HCl (pH 7.9), 1 mM EDTA, 0.5 mM NaCl, 0.1% bovine serum albumin, 0.1 mM spermidine and 5% glycerol) and the tested compounds. The mix was incubated for 15 min at 37 °C. Then, the reaction was initiated by addition of recombinant hTopo I (2 U), incubated at 37 °C for 1 h and terminated by the addition of 5× stop buffer (5% sarkosyl, 25% glycerol, 0.125% bromophenol blue). The aqueous phase was loaded onto a 1% agarose gel containing 1× TAE buffer (diluted from50× buffer containing 242 g Tris base, 57.1 mL glacial acetic acid and 100 mL of 0.5 M EDTA) without ethidium bromide (EB). At the end, 1× TAE buffer containing EB (0.5 μg/mL) was used to stain agarose gel for 30 min and after washing with distilled water for 15 min, it was visualized using a UV transilluminator.

Similarly, hTopo II decatenation assays were carried out, as indicated in the manufacturer’s procedures (TopoGEN, Port Orange, FL, USA) with some revisions [15].

hTopo II decatenation assays were performed in a final volume of 20 μL: 0.3 μg of kinetoplast DNA (kDNA) was added to a solution containing 1× assay buffer (50 mM Tris-HCl, pH of 8, 150 mM NaCl, 10 mM MgCl2, 0.5 mM Dithiothreitol (DTT), 30 μg/mL bovine serum albumin (BSA), 1 mM ATP) and the tested compounds. The mix was incubated for 15 min at 37 °C. Then, the reaction was started by adding 3 U of hTopo II and incubating at 37 °C for 1 h. Then, 5× stop buffer was added and the samples were treated as described in the previous paragraph. The aqueous phase was loaded on a 1% agarose gel containing 1× TAE buffer with EB (0.5 μg/mL) and visualized using an UV transilluminator.

#### 4.2.4. Immunofluorescence Analysis

The cells were plated and further processed, as previously indicated [30]. Specifically, the rabbit anti-β-Actin (Santa Cruz Biotechnology, Dallas, TX, USA), diluted 1:100 in bovine serum albumin (BSA) 2%, was used as primary antibody and incubated overnight at 4 °C. The secondary antibody, Alexa Fluor^®^ 488 conjugate goat-anti-rabbit, was diluted 1:500 and incubated for 2 h at 37 °C. DAPI (4′,6-diamidino-2-phenylindole, Sigma Aldrich, Milan, Italy) 0.2 μg/mL was used for nuclei staining. A fluorescence microscope (Leica DM 6000) was utilized for fluorescence detection (40× magnification). LAS-X software allowed acquiring and processing all the fluorescence images, which are representative of three separate experiments.

#### 4.2.5. Actin Polymerization/Depolymerization Assay

An Actin Polymerization/Depolymerization Assay Kit, purchased from Abcam, was employed to assess the ability of compounds **3** and **4** to interfere with the actin polymerization and depolymerization processes. To perform the essays, the manufacturer’s instructions were followed with some modifications [60]. In particular, polymerization assay was carried out incubating in a white 96-well plate the reconstituted actin with supplemented Buffer G, compounds **3** and **4** and then Buffer P was added in order to induce actin polymerization. For the Actin Depolymerization Assay, the actin polymerization was first induced, incubating supplemented Buffers P and G at room temperature for one hour. Then, compounds **3** and **4** were added at a concentration of 5 μM. Latrunculin A (LA) and Cytochalasin B (CB) were utilized as positive control at a concentration of 5 μM. For both the assays, the assemblage of the actin filaments was defined by measuring the fluorescence (Ex/Em: 365/410 nm) in kinetic mode for 1 h at room temperature in a microplate reader.

#### 4.2.6. Tunel Assay

TUNEL assay was employed to assess the cells apoptosis, following the manufacturer’s protocols (CF™488A TUNEL Assay Apoptosis Detection Kit, Biotium, Hayward, CA, USA) with few revisions. Briefly, the cells were plated and then additional processed as previously described [30]. DAPI (0.2 μg/mL, Sigma Aldrich, Milan, Italy) was used for nuclei staining. A fluorescence microscope (Leica DM 6000) was used for fluorescence detection (20× magnification). LAS-X software allowed acquiring and processing all the fluorescence images, which are representative of three separate experiments.

### 4.3. In-Silico Predictive Studies

The targeted molecules were appraised for predicting the drug-likeness based on 5 separate filters, namely Lipinski, Ghose, Veber, Egan, and Muegge [61,62,63,64,65,66] rules, accompanying bioavailability and drug-likeness scores obtained using the Molsoft software and SwissADME program (http://swissadme.ch, The access date was 10 January 2022) using the ChemAxon’s Marvin JS structure drawing tool.

## 5. Conclusions

Since the discovery of Ellipticine, carbazole derivatives have attracted the interest of the scientific world because of their versatility and wide range of applications. Herein, we described the interesting anticancer properties of a small series of carbazole derivatives observed using *in silico* and *in vitro* studies. The most active compounds **3** and **4** were found to be particularly active against the highly aggressive and metastatic MDA-MB-231 cells, without cytotoxicity on the normal counterpart. Further studies proved that they are able to selectively inhibit the hTopo I and actin polymerization, promoting, at the same time, the F-actin depolymerization. As a final and combined effect, both compounds induced apoptosis in the MDA-MB-231 breast cancer cells. Finally, these compounds deserve to be further developed as new multi-target agents in the treatment of triple negative breast cancer, currently characterized by a poor prognosis and for which few valid therapeutic options are available.

## Figures and Tables

**Table 1:** IC_50_ values of carbazoles derivatives and Ellipticine expressed in μM. The means ± standard deviations are shown.

IC_50_ (µM)			
Compounds	MDA-MB-231	MCF-7	MCF-10A
**1**	43.45 ± 1.21	>100	>100
**2**	8.19 ± 0.26	>100	89.16 ± 0.47
**3**	1.44 ± 0.97	27.58 ± 0.71	51.89 ± 0.88
**4**	0.73 ± 0.74	19.76 ± 1.12	>100
**5**	6.59 ± 0.68	>100	>100
Ellipticine	1.92 ± 0.38	1.34 ± 0.40	1.12 ± 0.51

**Table 2:** Binding free energies of carbazole derivatives against hTopo I, hTopo II and actin.

Compounds	hTopo I	hTopo II	Actin
**1**	−7.22	−8.13	−7.52
**2**	−6.46	−7.17	−7.56
**3**	−8.62	−7.61	−7.42
**4**	−7.95	−8.75	−8.0
**5**	−6.30	−7.75	−6.88

The binding energies are calculated using the software Autodock 4.0.2 and expressed in Kcal/mol.

**Table 3:** Drug likeness predictions of tested compounds.

No	MW	Number of HBA ^a^	Number of HBD ^b^	Log *P*_o/w_ (iLOGP) ^c^	Log S ^d^	TPSA ^e^	BBB Permeant ^f^	Lipinski, Ghose, Veber, Egan, and Muegge Violations	Bioavailability Score	Drug-Likeness Model Score
**1**	374.27	2	0	3.85	Poorly soluble	31.23	No	0	0.55	−0.43
**2**	325.40	3	0	3.73	Moderately Soluble	40.46	Yes	0	0.55	−0.47
**3**	339.19	4	2	0.00	Moderately Soluble	71.69	Yes	0	0.55	0.52
**4**	311.37	3	1	3.11	Moderately soluble	51.46	Yes	0	0.55	0.42
**5**	211.26	1	2	1.91	Moderately soluble	36.02	Yes	0	0.55	−0.15

(a) number of hydrogen bond acceptors; (b) number of hydrogen bond donors; (c) lipophilicity; (d) Water solubility (SILICOS-IT [S=Soluble]); (e) topological polar surface area (Å^2^); (f) Blood Brain Barrier permeant; lipinsky NorO > 10.

## Data Availability

Not applicable.

## Supplementary Materials

The following supporting information can be downloaded at: https://www.mdpi.com/article/10.3390/ph16030353/s1, Figure S1: The three-dimensional structure of the human proteins Topoisomerase I (Panel A), Topoisomerase II (Panel B) and Actin (Panel C) bound to compounds **1**, **2** and **5** are drawn. Proteins are schematically reported as ribbons. Ligands binding poses are described as colored sticks.; Table S1: The main calculated pharmacokinetic descriptors studied on pkCSM predictive models.
