# Supplementary material for: 5,8-Dimethyl-9H-carbazole Derivatives Blocking hTopo I Activity and Actin Dynamics
Source: Pharmaceuticals (Basel). 2023 Feb 25;16(3):353. doi: 10.3390/ph16030353 (PMC10051477; doi:10.3390/ph16030353)
Supplement: Supplementary file 1 [file pharmaceuticals-16-00353-s001.zip › pharmaceuticals-2183581-supplementary.pdf]

**Supplementary material**

**Figure S1**

**Table S1**

*Article*

# 5,8-dimethyl-9*H*-carbazole derivatives blocking hTopo I activity and actin dynamics

**Jessica Ceramella [1], Domenico Iacopetta [1,*], Anna Caruso [1], Annaluisa Mariconda [2], Anthi Petrou [3], Athina Geronikaki [3], Camillo Rosano [4], Carmela Saturnino [2], Alessia Catalano [5], Pasquale Longo [6] and Maria Stefania Sinicropi [1,]**

[1] Department of Pharmacy, Health and Nutritional Sciences, University of Calabria, 87036, Arcavacata di Rende, Italy
[2] Department of Science, University of Basilicata, 85100 Potenza, Italy
[3] Department of Pharmacy, School of Health, Aristotle University of Thessaloniki, 54124, Thessaloniki, Greece
[4] U.O. Proteomica e Spettrometria di Massa, IRCCS Ospedale Policlinico San Martino, Largo R. Benzi 10, 1632 Genova, Italy
[5] Department of Pharmacy-Drug Sciences, University of Bari "Aldo Moro", 70126 Bari, Italy
[6] Department of Chemistry and Biology, University of Salerno, Via Giovanni Paolo II, 132, 84084 Fisciano, Italy
* Correspondence: domenico.iacopetta@unical.it; Tel.: +39-0984-493200

## Panel A

1

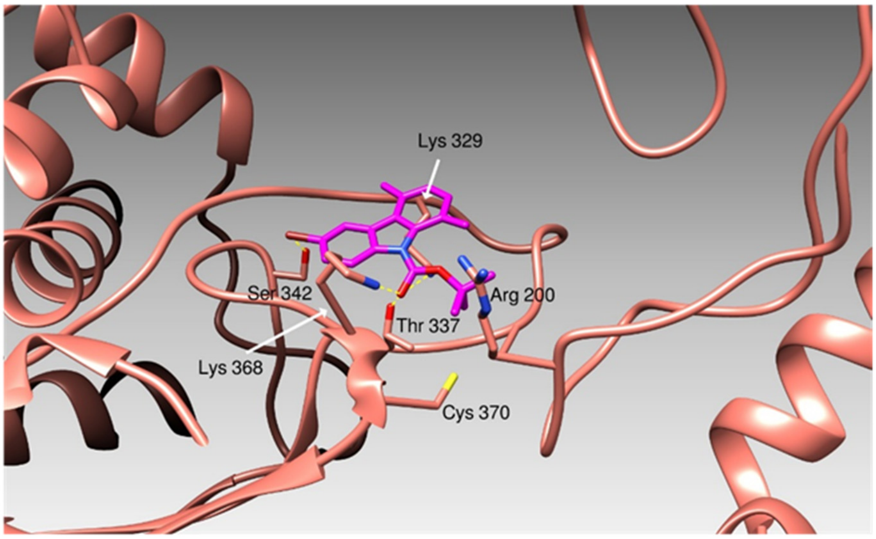
Lys 329
Ser 342
Arg 200
Thr 337
Lys 368
Cys 370

2

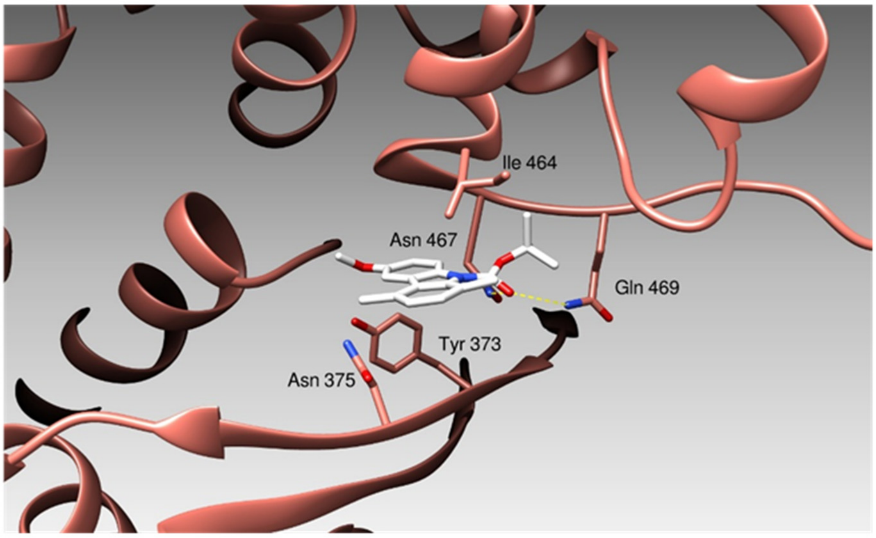
Ile 464
Asn 467
Gln 469
Tyr 373
Asn 375

5

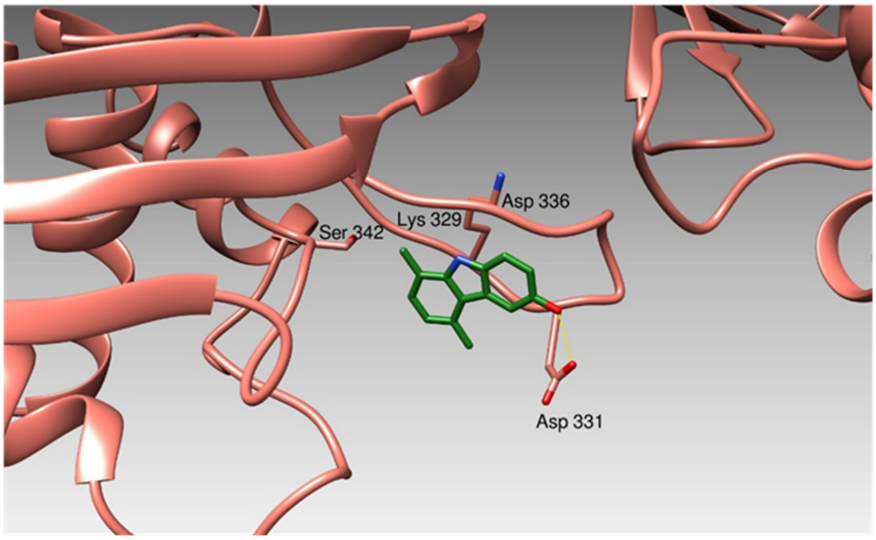
Asp 336
Lys 329
Ser 342
Asp 331

## Panel B

1

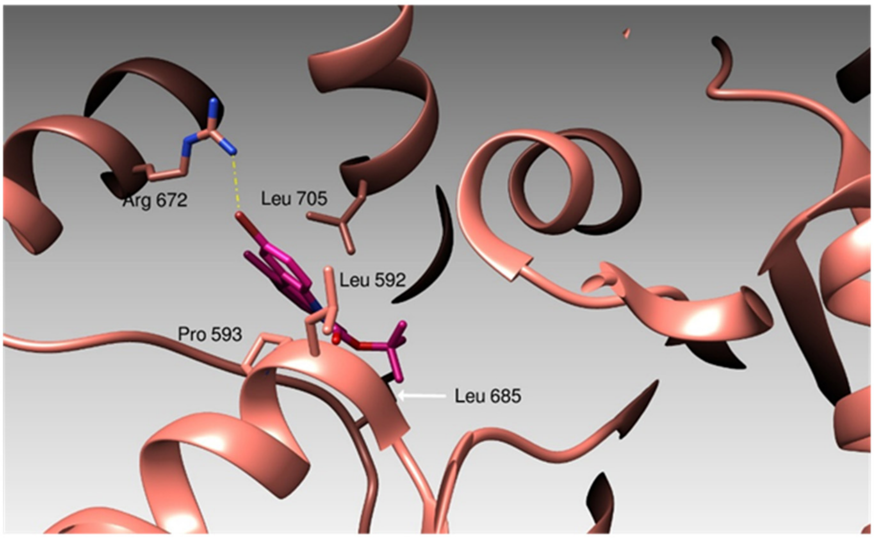
Arg 672
Leu 705
Leu 592
Pro 593
Leu 685

2

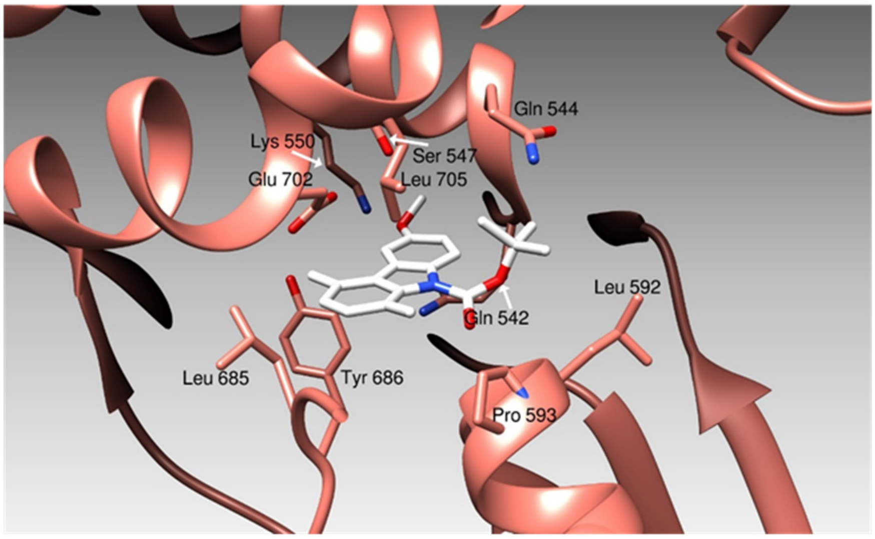
Gln 544
Lys 550
Ser 547
Glu 702
Leu 705
Leu 592
Gln 542
Leu 685
Tyr 686
Pro 593

5

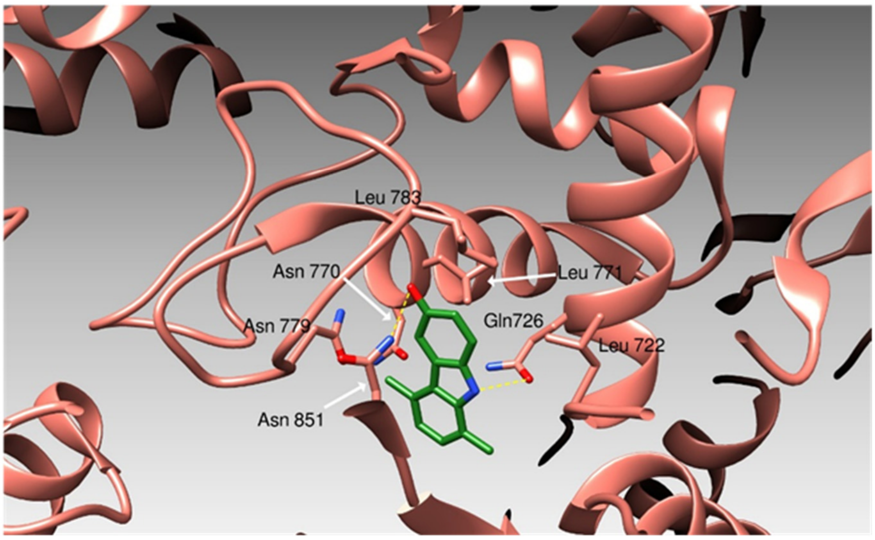
Leu 783
Asn 770
Leu 771
Gln726
Asn 779
Leu 722
Asn 851

**Panel C**

1

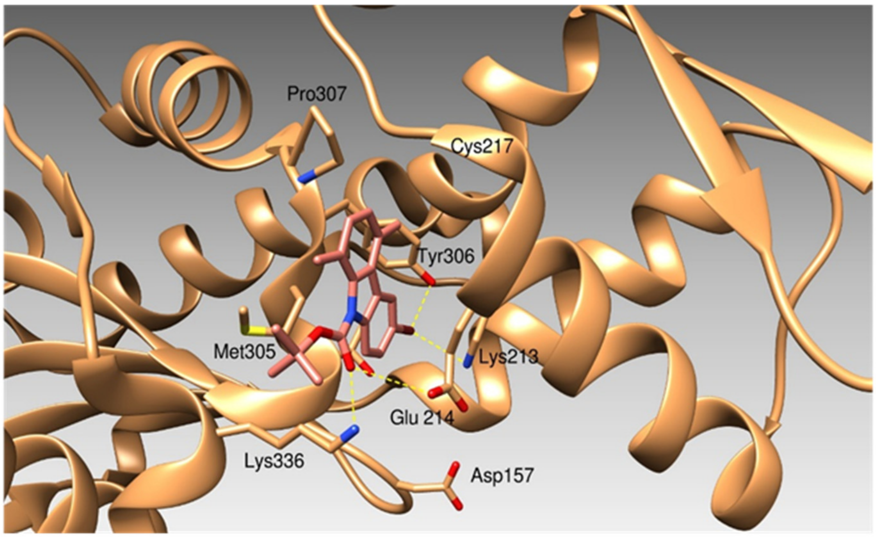
Pro307
Cys217
Tyr306
Met305
Lys213
Glu 214
Lys336
Asp157

2

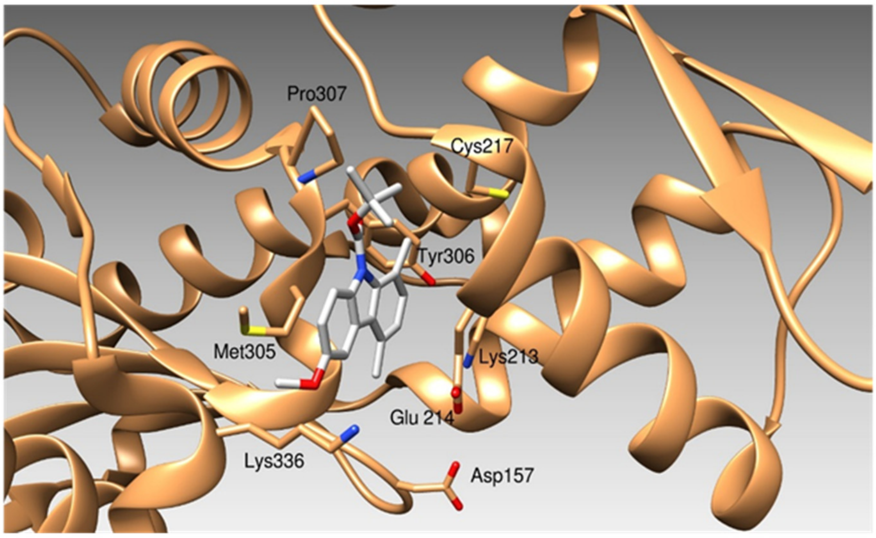
Pro307
Cys217
Tyr306
Met305
Lys213
Glu 214
Lys336
Asp157

5

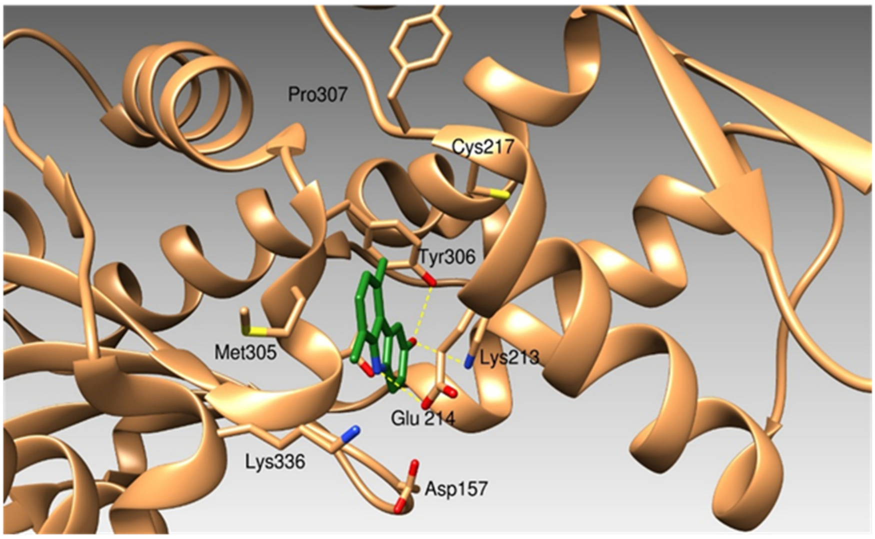
Pro307
Cys217
Tyr306
Met305
Lys213
Glu 214
Lys336
Asp157

**Figure S1.** The three-dimensional structure of the human proteins Topoisomerase I (Panel A), Topoisomerase II (Panel B) and Actin (Panel C) bound to compounds **1**, **2** and **5** are drawn. Proteins are schematically reported as ribbons. Ligands binding poses are described as colored sticks.

**Table S1**. The main calculated pharmacokinetic descriptors studied on pkCSM predictive models [33].

| No | Absorption | | | | Distribution | | Metabolism | |
|---|---|---|---|---|---|---|---|---|
| | Wsol | Caco2 | HIA | P-glycoprotein substrate | BBB | CNS | Cyp2d6s | CYP3A4s |
| **1** | -6.247 | 0.997 | 95.543 | No | -0.293 | -1.278 | No | Yes |
| **2** | -5.471 | 0.825 | 97.637 | No | -0.135 | -1.335 | No | Yes |
| **3** | -4.276 | 1.237 | 94.480 | No | -0.459 | -2.166 | No | Yes |
| **4** | -5.253 | 1.364 | 94.387 | No | 0.026 | -1.446 | No | Yes |
| **5** | -3.704 | 1.303 | 91.355 | Yes | 0.328 | -1.299 | No | Yes |

***WSol****, Water solubility in 25oC (mg/L);* ***Caco2****, permeability of Caco2 cell line (Papp in x10-6 cm/s) high permeability of Caco2 would translate in values > 0.90 ;* ***HIA****, human intestinal absorption (% Absorbed);* ***BBB****, represents the BBB permeability as logBB (the logarithmic ratio of brain to plasma concentrations)LogBB>0.3 cross the brain, while logBB<-1 is poorly distributed to the brain;* ***CNS****, blood-brain permeability-surface area product as (logPS) compounds with logPS>-2 are considered to penetrate the CNS;* ***CYP2D6s****, substrate for CYP450 isoform 2D6;* ***CYP3A4s****, substrate for CYP450 isoform 3A4.*

## References

[33] Pires, D.E.; Blundell, T.L.; Ascher, D.B. pkCSM: Predicting Small-Molecule Pharmacokinetic and Toxicity Properties Using Graph-Based Signatures. *J Med Chem* **2015**, *58*, 4066-4072.
